# Racial Disparities of Alcohol-Associated Liver Disease and Its Complications in Hospitalized Patients With Alcohol Use Disorder

**DOI:** 10.1016/j.gastha.2024.100592

**Published:** 2024-11-29

**Authors:** Soo Young Hwang, Elina Stoffel, David Sooik Kim, Elizabeth Verna, Wei Zhang

**Affiliations:** 1 Harvard T. H. Chan School of Public Health, Boston, Massachusetts; 2 Gastroenterology Unit, Massachusetts General Hospital, Harvard Medical School, Boston, Massachusetts; 3 Center for Liver Disease and Transplantation, New York-Presbyterian Columbia University Irving Medical Center, New York City, New York; 4 Department of Internal Medicine, Rutgers New Jersey Medical School, Newark, New Jersey

Alcohol use disorder (AUD), a major contributor to alcohol-associated liver disease (ALD), has been increasing in the United States. As a result, ALD, from alcohol-associated hepatitis (AAH) to alcohol-associated cirrhosis (AAC) and hepatocellular carcinoma, has become the leading cause of death from cirrhosis and the primary reason for liver transplantation.^1^

Therefore, it is imperative to understand the factors associated with the development of ALD in AUD patients. Racial disparities and associated factors such as differing access to health care, racial discrimination, and environmental factors have been suggested to play a significant role in the utilization of alcohol treatment. However, little is known about how racial disparities affect the development of ALD in AUD patients. Our research aims to identify the racial and ethnic disparities of ALD and its complications in patients hospitalized with AUD.

We retrospectively analyzed all patients over the age of 18 admitted at the New York Presbyterian/Columbia University Irving Medical Center, a large urban tertiary center between January 2010 and January 2020 who had AUD based on validated International Classification of Diseases 9 and 10 codes. We excluded all other forms of liver disease including hepatitis B and C, Wilson’s disease, hereditary hemochromatosis, and alpha-1 antitrypsin deficiency.

Data on race and ethnicity were retrieved and recoded into 5 categories based on the U.S. Census data^2^; non-Hispanic Asian, non-Hispanic African American, Hispanic, and other non-Hispanic races, with the non-Hispanic White population as the reference group due to its large sample size. Our main outcomes of interest were ALD such as AAH and compensated alcoholic cirrhosis, defined as the absence of hepatocellular carcinoma with cirrhosis, hepatic encephalopathy (HE), spontaneous bacterial peritonitis, variceal bleeding, and ascites. Occurrences of any one of the complications mentioned were subcategorized under decompensated cirrhosis.

Chi-square tests were performed to compare the proportions, and analysis of variance was used to compare the means of baseline characteristics. Multivariate logistic regression model, adjusting for year of admission, age, sex, marital status, diabetes mellitus, congestive heart failure (CHF), myocardial infarction (MI), chronic obstructive pulmonary disease, and chronic kidney disease, was built to compare the odds of developing ALD in different racial/ethnicity groups.

A total of 17,378 patients were analyzed: non-Hispanic White (25.6%), non-Hispanic Asian (1.6%), non-Hispanic African American (11.4%), and Hispanics (19.4%). The mean age was lower in non-Hispanic Asians (60.8 years) and non-Hispanic African Americans (60.7 years) and highest in non-Hispanic Whites (66.7 years). Hispanics had the highest proportion of patients with diabetes mellitus (60.6%) while non-Hispanic White patients had the highest proportion of MI (18.1%) and CHF (49.5%). Non-Hispanic Asians had the lowest of both (13.3% of MI and 35.6% of CHF). Non-Hispanic African American patients had the highest proportion of those diagnosed with chronic kidney disease (42.0%) (Table 1).

**Table 1:** Baseline Characteristics of the Study Population (n = 17,378)

Baseline characteristics	Non-Hispanic White (n = 4453)	Non-Hispanic Asian (n = 270)	Non-Hispanic African American (n = 1977)	Hispanics (n = 3732)	Others (n = 2049)
Age (y) mean (SD)	66.7 (15.5)	60.8 (14.3)	60.7 (15.6)	64.2 (15.6)	63.6 (15.8)
Female % (n)	34.7 (n = 1546)	33.0 (n = 89)	46.5 (n = 920)	43.7 (n = 1631)	39.6 (n = 812)
Marital status % (n)					
Single	27.5 (1226)	25.2 (68)	60.0 (1185)	51.2 (1910)	51.5 (1056)
Married	55.0 (2447)	64.1 (173)	24.7 (489)	30.8 (1150)	22.4 (459)
Divorced	4.6 (204)	4.8 (13)	4.4 (87)	5.4 (202)	3.5 (72)
Widowed	10.9 (484)	4.4 (12)	8.1 (160)	9.8 (364)	7.5 (154)
Other	2.1 (92)	1.5 (4)	2.8 (56)	2.8 (106)	15.0 (308)
Diabetes mellitus % (n)	43.2 (1925)	50.4 (136)	52.0 (1029)	60.6 (2263)	49.6 (1016)
MI % (n)	18.1 (806)	13.3 (36)	14.6 (288)	16.3 (609)	18.3 (374)
CHF % (n)	49.5 (2206)	35.6 (96)	45.8 (905)	39.9 (1488)	46.9 (960)
Chronic obstructive pulmonary disease % (n)	6.6 (293)	3.0 (8)	8.9 (175)	9.1 (341)	9.4 (193)
Chronic kidney disease % (n)	35.7 (1590)	31.1 (84)	42.0 (831)	34.9 (1302)	38.8 (796)

SD, standard deviation.

Our final multivariate logistic regression model adjusted for age, sex, marital status, diabetes mellitus, MI, CHF, chronic obstructive pulmonary disease, and chronic kidney disease is presented in Table 2. Hispanic subjects, compared to non-Hispanic White subjects, had higher odds of AAH (odds ratio (OR): 1.92, 95% confidence interval (CI): 1.43–2.59) (*P* value < .01), decompensated AAC (OR: 1.26, 95% CI: 0.97–1.64) (*P* value .09), and variceal bleeding (OR: 1.57, 95% CI: 1.08–2.27) (*P* value .02). Non-Hispanic Asians had lower odds of decompensated AAC compared to non-Hispanic Whites (OR: 0.34, 95% CI: 0.10–0.82) (*P* value .04). Non-Hispanic African Americans had lower odds of decompensated AAC in non-Hispanic Whites (OR: 0.65, 95% CI: 0.45–0.96) (*P* value .04) and lower odds of HE compared to non-Hispanic Whites (OR: 0.52, 95% CI: 0.36–0.73) (*P* value < .01).

**Table 2:** Odds of ALD and Its Complications in Hospitalized Patients With Alcohol Use Disorder According to Race

ALD	Non-Hispanic White	Non-Hispanic Asian	*P* value	Non-Hispanic Black/African American	*P* value	Hispanics	*P* value	Others	*P* value
		Odds ratio [95% CI]		Odds ratio [95% CI]		Odds ratio [95% CI]		Odds ratio [95% CI]	
Alcohol-associated Hepatitis	1	0.552 [0.164–1.388]	.263	1.398 [0.951–2.038]	.085	1.918 [1.428–2.589]	<.001	2.571 [1.827–3.623]	<.001
Compensated cirrhosis	1	0.427 [0.130–1.036]	.099	0.866 [0.597–1.234]	.436	1.063 [0.808–1.396]	.662	1.571 [1.159–2.124]	.003
Decompensated cirrhosis	1	0.337 [0.102–0.818]	.035	0.647 [0.421–0.964]	.039	1.259 [0.965–1.642]	.089	1.692 [1.245–2.290]	<.001
Hepatocellular carcinoma with cirrhosis	1	0.613 [0.098–2.061]	.507	0.973 [0.447–1.941]	.941	1.065 [0.632–1.778]	.811	1.601 [0.902–2.795]	.102
Hepatic Encephalopathy	1	0.595 [0.289–1.089]	.120	0.519 [0.363–0.726]	<.001	0.940 [0.747–1.180]	.594	1.267 [0.972–1.644]	.078
Spontaneous Bacterial Peritonitis	1	0.757 [0.263–1.722]	.553	0.810 [0.499–1.272]	.375	1.038 [0.728–1.473]	.835	1.663 [1.135–2.421]	.008
Variceal bleeding	1	0.784 [0.234–1.962]	.645	0.895 [0.509–1.512]	.689	1.566 [1.083–2.274]	.018	2.300 [1.473–3.570]	<.001
Ascites	1	0.506 [0.027–2.701]	.522	0.987 [0.448–2.096]	.972	0.726 [0.384–1.365]	.322	0.508 [0.072–2.103]	.414

Adjusted by year of admission, age, sex, marital status, and comorbidities (diabetes mellitus, MI, CHF, chronic kidney disease, chronic obstructive pulmonary disease).

Others include patients who chose “Other race” as a category and American Indian/Alaskan, Pacific Islanders due to their small sample size.

Patients with decompensated cirrhosis are those with cirrhosis and HCC or HE or spontaneous bacterial peritonitis or variceal bleeding or ascites.

Hispanic patients compared to non-Hispanic White patients in our study had higher odds of AAH, decompensated AAC, and variceal bleeding while non-Hispanic Asians and non-Hispanic African Americans had significantly lower odds of decompensated AAC.

Our results align with a recent meta-analysis of 25 studies that reported pooled risk ratios for ALD prevalence were 1.64 (95% CI: 1.12–2.39) for Hispanic individuals and 0.59 (95% CI: 0.35–0.87) for Black individuals, compared to White individuals.^1^ In a prior study in Sacramento, California, Hispanic patients presenting with ALD were more likely to be male, younger, obese, and diabetic compared to White/Caucasians.^3^ Our Hispanic population also had the highest percentage of patients with diabetes mellitus (60.6%) which could contribute to increased hepatic inflammation and increased risk of ALD.

Non-Hispanic African Americans in our study had statistically significantly lower odds of decompensated cirrhosis and HE. Non-Hispanic Asians also showed a trend of overall lower odds of ALD and its associated complications compared to the reference group and the absence of statistical significance is assumed due to the relatively small sample size.

Differences in racial/ethnic groups have various contributory factors including differences in drinking patterns and access and utilization of AUD treatment. Hispanic individuals had a higher proportion of binge drinking compared to other races, and White individuals had a higher proportion of heavy drinking, while Asian Americans had the lowest proportion in both indices.^4^ Utilization of AUD treatment is also complex as it is affected by social norms, perceived needs, perception of family and friends, and access to appropriate services in which disparities in race/ethnicity exist.^4^

Genetic predispositions in certain racial and ethnic populations were also suggested, although prior research emphasized more on metabolic dysfunction-associated fatty liver disease. The allele of Patatin-like phospholipase domain containing 3 gene (PNPLA3) (rs738409[G], encoding I148M) was strongly associated with increased hepatic fat and hepatic inflammation and was most commonly found in Hispanics while another allele of PNPLA3 (rs6006460[T], encoding S453I) was associated with lower hepatic fat content in African Americans.^5^ A genome-wide association study on individuals of European descent confirmed PNPLA3 (rs738409) and identified transmembrane 6 Superfamily Member 2 gene and membrane-bound O-acyltransferase 7 gene as risk loci for alcohol-related cirrhosis; all 3 loci having a role in lipid processing.^5^

Among the hospitalized AUD patients, Hispanics had higher odds of AAH, decompensated cirrhosis, and complications such as variceal bleeding while non-Hispanic Asians and non-Hispanic African Americans had lower odds of decompensated cirrhosis compared to non-Hispanic White patients.

## Supplementary materials

Table A1
