# Supplementary material for: Racial Disparities of Alcohol-Associated Liver Disease and Its Complications in Hospitalized Patients With Alcohol Use Disorder
Source: Gastro Hep Adv. 2024 Nov 29;4(4):100592. doi: 10.1016/j.gastha.2024.100592 (PMC11846918; doi:10.1016/j.gastha.2024.100592)
Supplement: Table A1 [file mmc1.docx]

**Supplemental Table 1. Baseline characteristics of the study population (n=17,378).**

|  | Non-Hispanic White  (n=4,453) | Non-Hispanic Asian  (n=270) | Non-Hispanic African American  (n=1,977) | Hispanics  (n=3,732) | Others  (n=2,049) |
| --- | --- | --- | --- | --- | --- |
| **Age (years)** mean (SD) | 66.7 (15.5) | 60.8 (14.3) | 60.7 (15.6) | 64.2 (15.6) | 63.6 (15.8) |
| **Female** % (n) | 34.7 (n= 1,546) | 33.0 (n=89) | 46.5 (n=920) | 43.7 (n=1,631) | 39.6 (n=812) |
| **Marital Status** % (n) |  |  |  |  |  |
| Single  Married  Divorced  Widowed  Other | 27.5 (1,226)  55.0 (2,447)  4.6 (204)  10.9 (484)  2.1 (92) | 25.2 (68)  64.1 (173)  4.8 (13)  4.4 (12)  1.5 (4) | 60.0 (1,185)  24.7 (489)  4.4 (87)  8.1 (160)  2.8 (56) | 51.2 (1,910)  30.8 (1150)  5.4 (202)  9.8 (364)  2.8 (106) | 51.5 (1056)  22.4 (459)  3.5 (72)  7.5 (154)  15.0 (308) |
| **Diabetes Mellitus** % (n) | 43.2 (1,925) | 50.4 (136) | 52.0 (1,029) | 60.6 (2,263) | 49.6 (1,016) |
| **Myocardial Infarction** % (n) | 18.1 (806) | 13.3 (36) | 14.6 (288) | 16.3 (609) | 18.3 (374) |
| **Congestive Heart Failure** % (n) | 49.5 (2,206) | 35.6 (96) | 45.8 (905) | 39.9 (1,488) | 46.9 (960) |
| **COPD** % (n) | 6.6 (293) | 3.0 (8) | 8.9 (175) | 9.1 (341) | 9.4 (193) |
| **Chronic Kidney Disease** % (n) | 35.7 (1,590) | 31.1 (84) | 42.0 (831) | 34.9 (1,302) | 38.8 (796) |
